# The dawn of relaxed phylogenetics

**DOI:** 10.1371/journal.pbio.3001998

**Published:** 2023-01-25

**Authors:** Jacob L. Steenwyk, Antonis Rokas

**Affiliations:** 1 Howards Hughes Medical Institute and the Department of Molecular and Cell Biology, University of California, Berkeley, Berkeley, California, United States of America; 2 Department of Biological Sciences, Vanderbilt University, Nashville, Tennessee, United States of America; 3 Vanderbilt Evolutionary Studies Initiative, Vanderbilt University, Nashville, Tennessee, United States of America

## Abstract

In 2006, a landmark study on relaxed phylogenetics was published in PLOS Biology. This Perspective discusses how relaxed phylogenetics has influenced the field of evolutionary biology by enabling researchers to investigate evolution’s tempo.

## Teaser

Tracing the history of evolution across time is a primary goal of evolutionary biology. The 2006 publication of a landmark study on relaxed phylogenetics in *PLOS Biology* enabled biologists to shed light on evolution’s tempo and shaped the future of evolutionary studies.

This article is part of the *PLOS Biology* 20th Anniversary Collection.

**Fig 1:**
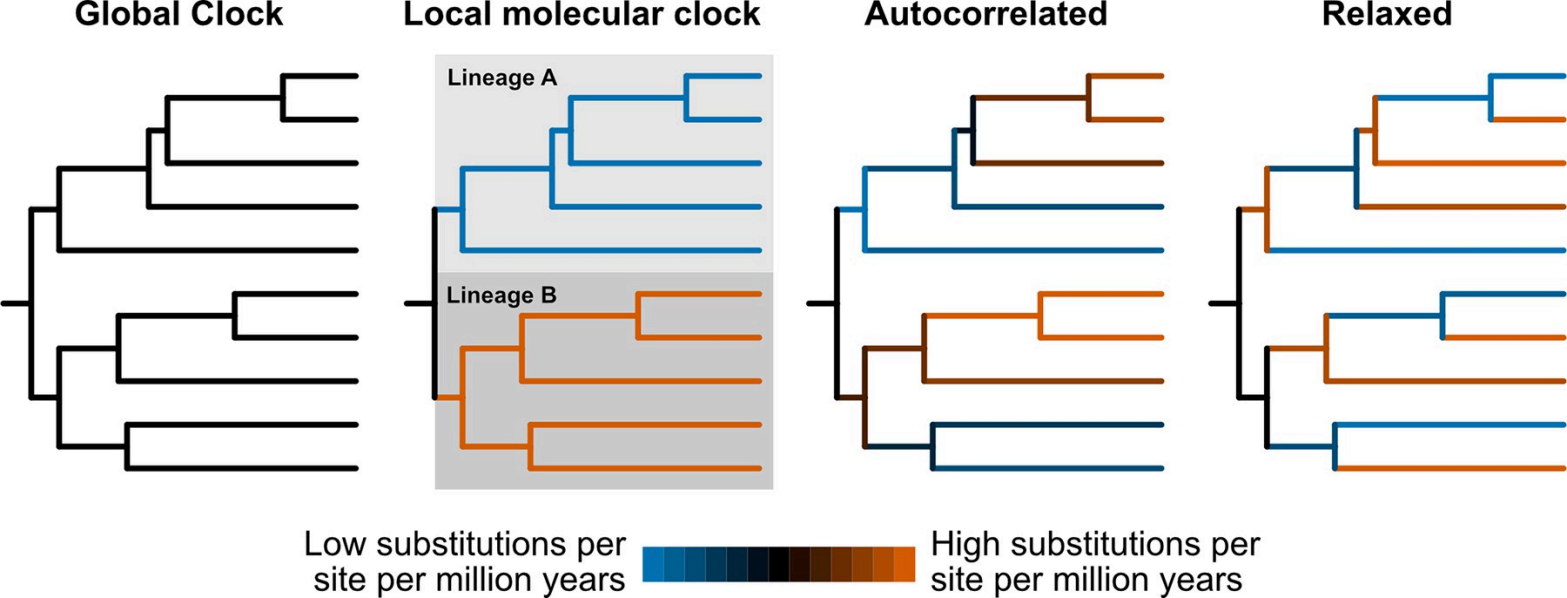
Cartoon representations of different clock models. Global clocks impose the same substitution rate across the phylogeny. Local clocks use the same substitution rate for user-defined lineages. Autocorrelated clocks assume that substitution rates gradually change across speciation events resulting in closely related lineages having similar rates. In relaxed clocks—the critical component of relaxed phylogenetics—the substitution rate of each branch is independent of other branches.

Since Emile Zuckerkandl and Linus Pauling proposed their hypothesis of a “molecular evolutionary clock” in their seminal 1965 paper titled “Evolutionary Divergence and Convergence in Proteins,” biologists have been fascinated by the prospect of adding a temporal dimension to their inferences of evolutionary relationships of genes and organisms. Early methods for divergence time estimation implemented a “global” model of clock-like evolution, which assumed a constant rate of sequence evolution along a fixed and presumed-to-be-correct phylogeny (Fig 1) [1]. However, it is now well known that rates of sequence evolution differ across lineages (e.g., rates of molecular evolution in organisms with shorter generation times, such as microbes, are much faster than those with longer generation times, such as mammals) violating a fundamental assumption in the global clock model. This variation in evolutionary rate can cause severe problems in molecular evolutionary dating and the inference of phylogenetic relationships [2]. Consequently, many statistical phylogenetic methods implement evolutionary models in which each branch has an independent rate of molecular evolution [3]. While these models revolutionized phylogenetic inference, they cannot disentangle evolutionary rate and time; a branch in a phylogenetic tree may be long (i.e., have a high number of substitutions) because the gene/organism in question is evolving at a fast pace or because it has been accumulating substitutions for a long time or both.

Given that rates of evolution vary, how can we then date phylogenies using molecular data? Early efforts to solve the challenge featured “local” molecular clocks that enabled users to define lineages that experienced different rates of sequence evolution (Fig 1) [4]. However, local clock models still require a known phylogeny and a priori knowledge of which lineages differ in their evolutionary rates. Autocorrelated relaxed clock models do not require a priori knowledge, implementing a model in which the rate of evolution can vary across branches in a phylogeny by positing that closely related species have similar rates and distantly related species may have different rates (Fig 1) [5]. However, autocorrelation of evolutionary rates is itself an assumption, is challenging to detect, and may vary depending on the breadth of taxon sampling and the evolutionary depth represented in the dataset. Moreover, constraining the phylogenetic tree can be problematic if branches are poorly supported. Lastly, converting relative divergence times to absolute times requires fixed calibration points; for example, fossils can provide a minimum age constraint but are susceptible to uncertainties such as placement on the phylogeny and absolute age.

In a landmark 2006 study in *PLOS Biology*, Drummond and colleagues developed a Bayesian Markov Chain Monte Carlo method to co-estimate phylogenies and divergence times that overcame the hurdles associated with earlier methods (Fig 1) [6]. Key features of this novel “relaxed” approach include allowing each branch in a phylogeny to have a different evolutionary rate (also known as the uncorrelated relaxed clock model), co-estimating substitution and relaxed clock parameters (e.g., substitution rates and time intervals), and implementing priors that took into account calibration uncertainties. These methods were incorporated in the foundational software BEAST [7].

Drummond and colleagues [6] accurately co-estimated phylogeny and divergence times from simulated and empirical data from diverse lineages to demonstrate the efficacy of relaxed phylogenetics. These analyses yielded numerous insights, such as the highly clock-like pattern of evolution among marsupial mammals. Divergence time estimation among marsupials also benefitted from using probabilistic priors, rather than fixed values, for calibration, thereby accounting for uncertainty in the fossil record. The relaxed phylogenetics approach also enabled measuring the clock-likeness of genes, revealing that clock-like evolution is relatively rare and that most genes exhibit non-clock-like rates of sequence evolution.

Relaxed phylogenetics ushered phylogeny estimation into the era where co-estimating phylogeny and divergence times could be done accurately and efficiently. Notable successes, which leverage present-day and ancient samples, include tracing the epidemiology of pathogenic viruses involved in notorious pandemics, like HIV [8], unraveling the timing of the evolution of charismatic megafauna, such as bison [9], and charting the course of recent human evolution [10]. Examining the evolutionary history of HIV shed light on its origin and transmission, facilitating the estimation of effective reproductive numbers in different geographic regions. For example, the relaxed phylogenetics approach revealed that the HIV-1 subtype B in the United States of America was likely introduced from the Caribbean in the early 1970s and underwent subsequent rapid expansion such that by the late 1970s the epidemic had already spread and diversified across the US [8]. Examination of bison evolution also benefitted from other features in BEAST, such as co-estimating demographic parameters (e.g., population growth and size) and divergence times [9]. Evolutionary relationships among Neanderthals, Denisovans, and modern humans were inferred using BEAST, helping elucidate human evolution [10], a pioneering achievement recently recognized in Svante Pääbo’s 2022 Nobel Prize in Physiology or Medicine. These three examples—chosen to highlight the power of relaxed phylogenetics—represent only a tiny sample of the seminal evolutionary insights that continue to be made using this method.

Future iterations of the relaxed phylogenetics approach will hopefully speed up computation, enabling use among increasingly large phylogenomic data matrices and reducing the environmental cost of extensive computation. Also, clarifying circumstances when the accuracy and precision of phylogenetic inference would improve from the use of a relaxed molecular clock approach may help guide experimental design and reduce computational burdens if models with greater complexity are not needed. Machine learning, other forms of artificial intelligence, and advances in computer science and engineering hold promise to overcome these issues.

The Swiss army knife-like properties of the BEAST software have also empowered evolutionary biologists and served as a pillar in the community. The success of BEAST is in part because the software is user-friendly, well-developed, has extensive and clear documentation, and is continually being updated. Companion software such as BEAUti, Tracer, and FigTree, among others, augment the BEAST ethos. These rich resources have enabled generations of scientists to conduct evolutionary inferences and help unravel the tempo and mode of biological evolution.

In the early days of the molecular evolutionary clock, model assumptions constrained the accuracy of divergence time estimates. By introducing relaxed phylogenetics, the 2006 *PLoS Biology* article by Drummond and colleagues enabled the widespread adoption of a biologically more realistic and accurate approach for estimating the tempo and mode of genetic evolution, facilitating impactful—including Nobel-worthy—discoveries.
